# The first year in formal schooling improves working memory and academic abilities

**DOI:** 10.1016/j.dcn.2023.101205

**Published:** 2023-01-29

**Authors:** Christina Davidson, Yee Lee Shing, Courtney McKay, Eva Rafetseder, Sobanawartiny Wijeakumar

**Affiliations:** a School of Psychology, University of Nottingham, Nottingham, United Kingdom; b Department of Psychology, Goethe University Frankfurt, Germany; c Center for Individual Development and Adaptive Education of Children at Risk (IDeA), Frankfurt, Germany; d Psychology, Faculty of Natural Sciences, University of Stirling, Scotland, UK

**Keywords:** Schooling, FNIRS, Vocabulary, Working memory

## Abstract

Neurocognition and academic abilities during the period of 4 and 7 years of age are impacted by both the transition from kindergarten to primary school and age-related developmental processes. Here, we used a school cut-off design to tease apart the impact of formal schooling from age, on working memory (WM) function, vocabulary, and numeracy scores. We compared two groups of children with similar age, across two years: first-graders (FG), who were enrolled into primary school the year that they became eligible and kindergarteners (KG), who were deferred school entry until the following year. All children completed a change detection task while brain activation was recorded using portable functional near-infrared spectroscopy, a vocabulary assessment, and a numeracy screener. Our results revealed that FG children showed greater improvement in WM performance and greater engagement of a left-lateralized fronto-parietal network compared to KG children. Further, they also showed higher gains in vocabulary and non-symbolic numeracy scores. This improvement in vocabulary and non-symbolic numeracy scores following a year in primary school was predicted by WM function. Our findings contribute to a growing body of literature examining neurocognitive and academic benefits conferred to children following exposure to formal schooling.

## Highlights

• The study aimed to tease apart effects of formal schooling from maturation.
• Similarly-aged first-graders were compared with kindergarteners across two years.
• First-graders showed better working memory, vocabulary and numeracy scores.
• First-graders showed greater left fronto-parietal engagement compared to kindergarteners.
• Working memory function was linked to better vocabulary and numeracy scores.

## 1 Introduction

Children between 4 and 7 years of age undergo a dynamic shift in neurocognitive functions and academic abilities. It is unclear to what extent this shift represents the transition from a kindergarten environment to a formal school learning environment because it is simultaneously confounded by age-related developmental changes. Thus, to isolate the true impacts of formal schooling on cognitive and brain development, it is important to disentangle schooling-related effects from age-related effects. From a pedagogical perspective, the more we understand the benefits and challenges of how formal schooling as an environment impacts neurocognitive development (and how it relates to academic abilities), the more we can shape practices to help bridge and smoothen children’s transition from kindergarten to school. This information might also be important for parents who live in countries that adopt an arbitrary cut-off date for school entry. These parents must decide whether to enrol their children in school as soon as they become eligible or defer their entry for the following year. Thus, it might be important to understand whether exposure to formal schooling leads to better, unchanged, or reduced neurocognitive function and academic abilities.

In previous work, quasi-experimental approaches such as school cut-off designs have been used to disentangle schooling-related effects from age-related effects (Morrison et al., 2019). In school cut-off designs, two groups of children are compared; one group whose birthday falls several weeks before the school enrolment cut-off and whose parents have decided to enrol them in primary school in the same year and another group whose birthday falls several weeks after the cut-off and whose parents have decided to defer school entry until the following year. Thus, both groups of children are similar in age, but differ in their experience of attending primary school. When followed across time and compared, it is then possible to tease apart the impact of schooling from age-related effects. Collectively, previous work employing school cut-off designs has demonstrated that the exposure to the formal schooling is associated with improvements in literacy (Varnhagen et al., 1994, Christian et al., 2000, Morrison et al., 1995, Kim and Morrison, 2018, Morrison et al., 1997, Ferreira and Morrison, 1994) and mathematics (Bisanz et al., 1995). However, there is limited evidence demonstrating the impact of schooling on executive function (Brod et al., 2017, Burrage, 2008, Kim et al., 2021, McKay et al., 2021). Brod et al. (2017) conducted the first-ever study comparing executive functions between first-graders and kindergarteners using a cut-off design. In this study, children completed a cognitive control task and an inhibitory control task. The authors reported that first-graders performed better than kindergarteners on the cognitive control task. Further, in first-graders, posterior parietal activation during the inhibitory control task was correlated with performance on the cognitive control task. Thus, the authors suggested that, in first-graders, increased engagement of the posterior parietal cortex, an area important for sustained attention, reflected how brain mechanisms might have become shaped by exposure to a formal schooling environment (Brod et al., 2017). More recently, in our own research work conducted in Scotland, we investigated whether exposure to formal schooling impacted inhibitory control and response monitoring. There were no schooling-related effects on inhibitory control. However, children who had attended a year in primary school demonstrated a greater change in activation in the bilateral frontal cortex, related to response monitoring. Further, this change in frontal activation was positively associated with mathematics performance (McKay et al., 2021).

In the current study, we posit that another critical executive sub-function, working memory (WM), responsible for storing and manipulating information for more complex cognitive processes, might be impacted by exposure to formal schooling. In school, children will need to depend upon WM function to follow directions, interact with peers, actively maintain knowledge, keep track of routines and tasks and not succumb to distraction (Diamond, 2013, Finch, 2019). Improvements in WM performance in school children has been linked to time spent in school classrooms, above and beyond chronological age (Roberts, 2015). In older school children, WM performance is also related to improvements in academic skills such as numeracy and vocabulary (Gathercole et al., 2004, Gathercole and Pickering, 2000, Engel de Abreu and Gathercole, 2012). For instance, between the ages of 7 and 14, children with poor performance in WM tasks generally performed below the expected standards in national curriculum assessments of mathematics and English (Gathercole and Pickering, 2000, Gathercole et al., 2004). Bull et al. (2008) reported that visual-spatial short-term memory span was a significant predictor of mathematics outcomes for the first year of primary school. Similar associations between WM processing and vocabulary have been reported in pre-school and school-aged children (Engel de Abreu and Gathercole, 2012, Kim et al., 2015, Verhagen and Leseman, 2016). Gathercole et al. (1992) conducted a 4-year longitudinal study with children aged 4–8 and reported that WM at 4-years-old was significantly associated with vocabulary at 5-years-old. Furthermore, meta-analyses and computational modelling work suggests that WM function might be closely associated with inhibitory control and cognitive control, thus it is pertinent to assess whether previous schooling-related findings from Brod and colleagues might also reflect WM processing (Niendam, 2012, Wijeakumar et al., 2017).

The current study used a modified school cut-off design set within the Scottish schooling curriculum to tease apart schooling-related effects from age-related effects. In this curriculum, the school year commences in the month of August and the cohort is composed of children born between the months of March of the previous year (around 5.5 years of age at enrolment) and February of the same year (around 4.5 years of age at enrolment). On average, children are around 5 years of age at enrolment. Notably, parents of children born in January and February can choose to either enrol their child to begin school in August or defer their entry until the following year. Our research inquiry recruited and followed these two groups of children across two years. Thus, these children are similar in age but differed in their exposure to formal schooling: first-graders (FG) whose parents had made the decision to enrol their children into school the year they became eligible and kindergarteners (KG) whose parents had made the decision to defer school entry and allow them to stay in kindergarten for another year. Thus, comparing these two groups of children across time, we were able to extract schooling-related effects from age-related effects. We conducted all testing and assessments in children’s homes to allow more families to participate in the study. We used a color change detection task to assess WM function in children. This task has been previously used in adults and children to measure WM capacity and accuracy across increasing loads (Buss et al., 2014, Simmering, 2012, Ambrose et al., 2016, Wijeakumar et al., 2017). We used a portable functional near-infrared spectroscopy (fNIRS) system to measure brain function while children engaged with the task. Children also completed vocabulary and numeracy assessments and parents completed self-reported questionnaires.

The current study posed three research questions. Our first question inquired whether exposure to the first year of schooling improved WM function, above and beyond age-related effects. Based on evidence from Roberts et al. (2015), we predicted that FG, who had a year in school would show greater WM performance compared with KG children, who had been deferred entry for a year (Roberts, 2015). We expect that greater WM performance will be supported by greater engagement of regions in the fronto-parietal network. Here, we predicted that FG would show greater engagement of the fronto-parietal network compared to KG children. Our second question inquired whether one year of schooling would lead to greater improvement in vocabulary and numeracy scores, over and above age-related effects. Based on more general previous findings of improved math and literacy abilities in primary school children (Varnhagen et al., 1994, Christian et al., 2000, Kim and Morrison, 2018, Morrison et al., 1997), we predicted that FG children would demonstrate greater improvements in both scores compared to KG children. Our third question followed previous predictions to inquire whether any schooling-related improvements in WM function would be associated with any schooling-related improvements in academic abilities. Based on previous findings in school children, we predicted that gains in WM function would be linked to gains in academic skills (Gathercole et al., 2004, Engel de Abreu and Gathercole, 2012).

## 2 Materials and methods

### 2.1 Participants

Ethical approval was granted by the General University Ethics Panel at the University of Stirling - Approval Reference: GUEP 375 and GUEP 375(A). Participants were recruited through liaison with nurseries and primary schools across the central belt of Scotland. Information packs were distributed to parents of children born in January or February 2014. Following this, interested parents contacted the research team and potential participants were screened to ensure that they met the inclusion criteria: children had normal or corrected-to-normal vision, a normal delivery term (37–42 weeks), no exposure to drug or alcohol use during pregnancy, no family history of mental illness, no neurological conditions, no color blindness linked to themselves or relatives, and spoke English as their primary language.

Parents were required to declare if they had enrolled their child in Grade 1 the same year or had deferred school entry until the following year, so that we could categorize them into FG and KG children respectively. Informed consent was obtained from parents and assent obtained from their children prior to the commencement of the study. Power analysis assuming a medium effect size (estimated from (Brod et al., 2017)) and power of 0.95 suggested that 32 participants in each group was sufficient for detecting interaction between schooling group and time in a repeated measures ANOVA design.

For T1, data was collected between the months of May and September in 2018. Ninety-five 4.5-year-olds (45 females, *Mage* = 53.5 months, *SD* = 1.2) were recruited into the study. Data from 21 children were excluded from analyses: five refused to engage with the task, twelve interfered with the neuroimaging set-up by removing the cap, two had thick hair that prevented good contact between the optodes and scalp, and data from a further two children had to be excluded due to experimenter error. The final sample for T1 consisted of 37 FG (23 females, *Mage* = 53.7 months, *SD* = 1.4) and 37 KG (14 females, *Mage* = 53.3 months, *SD* = 1.2).

For T2, data was collected between the months of May and September in 2019. Data from 16 children (out of the 95 recruited into the study) were excluded from analyses: fifteen refused to engage with the task and data from one child had to be excluded due to experimenter error. The final cohort at T2 consisted of 40 FG (24 females, *Mage* = 65.7 months, *SD* = 1.1) and 39 KG (14 females, *Mage* = 65.3 months, *SD* = 1).

Note that at T1, all children were still in kindergarten. FG were enrolled to begin Grade 1 in September 2018 and KG were enrolled to begin Grade 1 in September 2019. Thus, at T2, FG had completed Grade 1 and KG had completed another year in kindergarten. Parents filled in a socioeconomic scale that assessed educational qualifications and annual income, and Strengths and Difficulties questionnaire (Goodman, 1997) that assessed children’s socio-emotional behaviours. There were no differences in socio-emotional behaviours or socioeconomic information between both groups. Further details are presented in the Supplementary Materials.

### 2.2 WM task

**Fig. 1:**
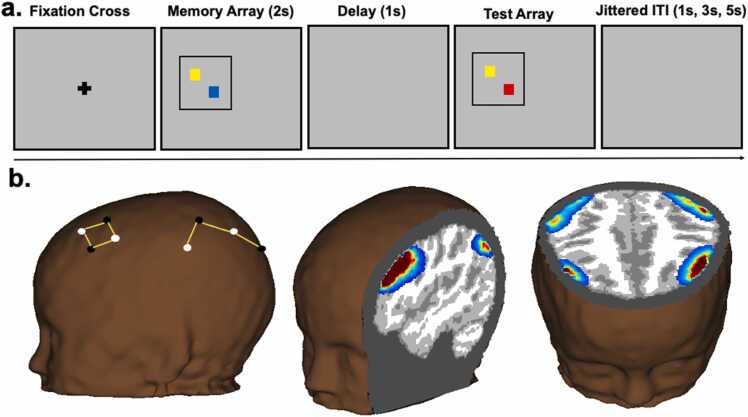
(a) Color change detection task – load 2 different trial (b) Probe geometry and sensitivity profiles after running Monte Carlo Simulations with 1 million photons on a 4.5-year-old atlas (white circles represent sources and black circles represent detectors).

WM performance was assessed using the color change detection task. We chose this task for a few reasons. It is relatively easy to explain the instructions to and engage pre-school children. Further, it does not use salient stimuli that can bias attention. Lastly, we have previously used this task and obtained reliable and reproducible data in children, young and older adults (Simmering, 2012, Wijeakumar et al., 2017, Ambrose et al., 2016). The experimenter explained the task to children using 3 × 3 in. flashcards with colored squares. The child was shown the first flashcard (one colored square) for approximately 2 s and was asked to remember the card. The first flashcard was then turned over and the second (a same or different square) was shown. The child was then asked whether the two cards were the same or different. Corrective feedback was given if necessary. A further two practice presentations were carried out, with each presentation increasing in WM load. Once all practice flashcards were successfully completed, the task was run in E-prime V.3 software on a HP laptop with a 14-inch screen. The task started with another 3 practice trials, where corrective feedback was given if required, before moving onto the experimental trials. Each trial began with a memory array of colored squares presented for 2 s, followed by a 1 s delay, and then ended with the test array of colored squares (Fig. 1a). The test array remained on the screen until children gave a response (same or different), which the experimenter recorded on the laptop. During “same” trials, colored squares in both arrays were identical. During “different” trials, one colored square was different in the test array. Before subsequent trials, there was an inter-trial interval of either 1 s (50%), 3 s (25%), or 5 s (25%). In every trial, memory and test arrays occupied the same position on the screen. However, across trials, arrays were presented on alternate sides to clearly distinguish trials. Loads were classified into low, medium, and high at each of the time-points (at T1 when participants were 4.5 years: low = 1 item, medium = 2 items, and high = 3 items and at T2 when participants were 5.5 years: low = 2 items, medium = 3 items, and high = 4 items). Each load featured randomised presentations of 8 same and 8 different trials. WM loads were increased from T1 to T2 to follow age progression and prevent ceiling effects (Simmering, 2012). Children had to complete the full run of the task to be included for the analyses.

### 2.3 fNIRS data acquisition

A NIRSport portable device with 8 sources and 8 detectors was used to measure brain activation during the WM task. Fibre optic cables transported infrared light from the fNIRS device to a tailored cap designed to collect HbO and HbR concentration. Four cap sizes (50 cm, 52 cm, 54 cm, and 56 cm) were used to accommodate varying head sizes. Source-detector separation was scaled according to cap size (50 cm: 2.5 cm; 52 cm: 2.6 cm; 54 cm: 2.7 cm; and 56 cm: 2.8 cm). Data was collected at 7.81 Hz using wavelengths of 860 nm and 750 nm. Potential positions for sources and detectors based on the 10–20 System of Electrode Placement were already indicated on the fNIRS caps (from the manufacturers). From these positions, a subset were chosen such that the channels would overlay the frontal and parietal cortices implicated in previous WM studies (Todd and Marois, 2004, Pessoa and Ungerleider, 2004). The positions were chosen such that the final probe geometry consisted of 14 channels, with 8 channels covering the frontal cortex (2 channels each covering right and left middle frontal gyrus and inferior frontal gyrus) and 6 covering the parietal cortex (one channel each covering left and right inferior parietal lobule, superior occipital gyrus, and supramarginal gyrus) - see Fig. 1b.

### 2.4 Numeracy assessment

Numeracy skills were tested using a numeracy screener (Nosworthy et al., 2013). For this task, children were required to compare magnitude pairs ranging from 1 to 9 and determine the larger of the two numbers. These magnitudes were represented with 56 symbolic (digits) and 56 non-symbolic pairs (dot arrays). In both conditions, the location where the larger digit or dot array appeared was counterbalanced. Additionally, dot arrays were controlled for area and density. After a short practice, children were given one-minute to complete each condition, with easier pairs preceding more difficult pairs. The presentation order of symbolic and non-symbolic items was counterbalanced. Children could achieve a score out of 56 for each condition. A single score was calculated for each condition, time-point, and participant by subtracting the number of incorrect responses from the number of correct responses.

### 2.5 Vocabulary assessment

Word knowledge was assessed using the Vocabulary subset of the Wechsler Preschool and Primary Scale of Intelligence (Warschausky and Raiford, 2018). This featured 3 picture items and 20 word items. Children were first shown a picture of a car, a banana, and a pair of scissors and asked to name each object. The child was corrected if they incorrectly named the first item (car). Feedback was not provided for the remaining picture items. For the word items, children were asked to provide verbal definitions of an orally presented word. Children were corrected on the first two items (sock, telephone) if they gave incorrect responses, but feedback was not provided for the remaining word items. Following the manual, if children’s responses were vague or unclear, the experimenter prompted them with neutral queries, such as “can you tell me anything else about …?”. The test was discontinued if there were 3 consecutive incorrect responses. Picture and verbal items were summed to provide a score. Thus, a single score was obtained for each participant at T1 and T2.

### 2.6 Procedure

Data was collected from children in their homes. Researchers explained the full procedure to the parent and obtained informed consent. Then, a quiet and spacious area was identified to set up the fNIRS and other testing equipment. After this, the child was seated on a chair and their head circumference was measured. The corresponding fNIRS cap size was selected, and sources and detectors were inserted into the cap. The child was given an iPad© to watch a short movie while two researchers fitted the cap on their head. The distances between the left and right peri-auricular points and inion and nasion were measured to correctly align the vertex of the cap with the centre of the head. A Polhemus Patriot motion sensor was used to digitize positions of scalp landmarks and sources and detectors while the children watched a cartoon. Unfortunately, due to problems with the frame of references, we could not use these digitisations. Thus, we obtained a template set of points for each of the four cap sizes that were used in the study (i.e., 50 cm, 52 cm, 54 cm and 56 cm). To do this, we digitized points on one child who had the appropriate head size for each cap. At time-point 1, 18 children fit a 50 cm cap, 53 children fit a 52 cm cap, one child fit a 54 cm cap and two children fit a 56 cm cap. At time-point 2, 11 children fit a 50 cm cap, 37 children fit a 52 cm cap, 28 children fit a 54 cm cap and three children fit a 56 cm cap. Next, one experimenter began the WM task by introducing it as “the color game” and explained the rules using flashcards. Brain activation was recorded as children completed the experimental task. Stickers were awarded once they completed each load, regardless of performance, to sustain their motivation. Once the WM task was completed, children were given the iPad© again while the experimenters removed the cap. Following a short break, children completed the vocabulary and numeracy assessments. Stickers were awarded after children completed each task. All children were remunerated with £ 10 and a toy upon completion of each time-point.

### 2.7 WM behavioural analyses

The average number of included trials was 47.7 (out of 48 trials). Accuracy and capacity were calculated from hits (H) and false alarms (FA) based on behavioural responses. Accuracy refers to how well a child performed during the task. Capacity represents the highest number of items a child could successfully recall.

The following formulae were used to calculate accuracy. The variants presented below, based on Simmering (2016) (Simmering, 2016), account for conditions when H > =FA and H < FA. For cases where H and FA were equal, accuracy was set to 0.5. Here, accuracy of 1 illustrated perfect performance and accuracy of 0.5 illustrated chance performance.

If H ≥ FA: Accuracy = ½ + { [ (H – FA) * (1 + H – FA)] / [4 * H * (1 – FA)]}

If H < FA: Accuracy = ½ - { [ (FA – H) * (1 + FA – H)] / [4 * FA * (1 – H)]}

To establish which loads showed a significant schooling group x time-point interaction, a repeated measures ANOVA with within-subject factors of load (low, medium, and high) and time-point (T1 and T2) and a between-subjects factor of schooling group (FG and KG) was run. There was a significant interaction between time-point and schooling group (*F* (1,69) = 6.35, *p* = .014, $\eta p^{2}$ = 0.084). The interaction between load, schooling group and time-point was not significant. Thus, accuracy estimates across low, medium, and high loads were averaged to create an average accuracy estimate for each participant and time-point. These estimates were used in further modelling analyses described below.

Capacity was calculated for each load using Pashler's (1988) formula below. The maximum value was used in further modelling analyses.

Capacity = Load * (H – FA) / (1 – FA)

### 2.8 fNIRS pre-processing

fNIRS pre-processing was done in *EasyNIRS* using HOMER2. Raw data was pruned to remove noisy channels and intensity was converted to optical density units (dRange = 0.01–300, SNRthresh = 2, SDrange = 0–45). Principal components analyses were conducted to identify and remove motion artifacts using *hmrMotionCorrectPCArecurse* (tMotion =1, tMask =1, STDEVthresh = 50, AMPthresh = 0.5, nSV = 0.97, maxIter =5). Following this, the data were scanned again for motion artifacts using *hmrMotionArtifactByChannel* (tMotion =1, tMask =1, STDEVthresh = 50, AMPthresh = 0.5). Stimulus markers within specified windows of uncorrected artifacts were removed using *enStimRejection* (tRange = −1 12). This window was chosen to capture any motion during the memory array, delay, test array, response and jittered ITI for each trial. The data were band-pass filtered using *hmrBandpassFilt* (hpf = 0.016, lpf =0.5).

### 2.9 fNIRS image reconstruction

**Fig. 2:**
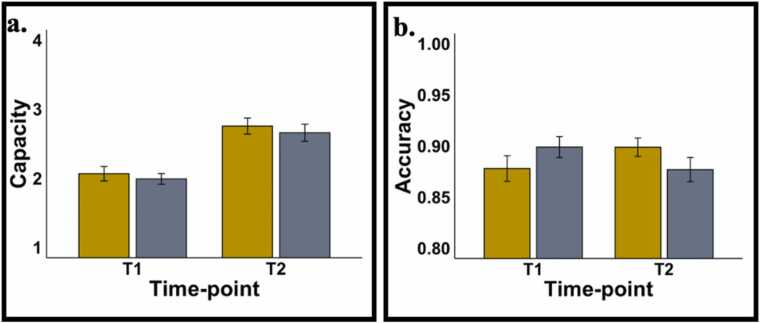
(a) Accuracy and (b) capacity at T1 and T2 for both groups. FG shown in yellow and KG showed in grey.

The methodological pipeline used in the current study has been described in detail elsewhere (Forbes et al., 2021). Here, we briefly outline each of the steps. A 4.5-year-old MRI atlas (to represent age at T1) and a 5.5-year-old MRI atlas (to represent age at T2) were obtained from the Neurodevelopmental MRI database (Richards et al., 2016) and segmented into four tissue types (scalp, cerebro-spinal fluid, grey matter and white matter). The digitized points for each cap size was projected onto both segmented head volumes and Monte Carlo simulations with 1 million photons were run to generate sensitivity profiles for each channel per head volume (4 cap sizes) and atlas (2 time-points) using *AtlasViewerGUI* in HOMER2 (shown in a 52 cm 4.5-year-old head in Fig. 2b). The head volumes and sensitivity profiles were then converted to NIFTI format. Optical density time-series data were integrated with these volumetric sensitivity profiles using a novel image reconstruction technique (Forbes et al., 2021, Eggebrecht, 2014) to create voxel-wise time-series data for each voxel, participant, and time-point. A general linear model with 12 regressors (3 loads [low, medium and high] x 2 trial types [same and different] x 2 accuracies [correct and incorrect]) was run on each voxel and time-point by convolving a modified gamma function from the SPM toolbox (delay of response = 4; delay of undershoot = 15; dispersion of response = 1; dispersion of undershoot = 1; ratio of response to undershoot = 6; onset =0; length of kernel =16) with a boxcar of duration 4 s. This boxcar window was chosen to account for a sample array presentation of 2 s, delay period of 1 s and 1 s of the test array presentation (see Fig. 2a for a schematic of each trial of the task). Beta coefficient maps obtained for each load, trial type, accuracy, participant, time-point, and chromophore were then registered to the MNI space. A group mask was constructed by summing beta maps from four representative participants (representing each of four head size templates and then, excluding voxels that contained data from less than 60% of the four templates. This group mask was used in the cluster-based thresholding below. Finally, a linear mixed effects model with within-subjects factors of load [low, medium and high], trial type [same and different], chromophore [HbO and HbR] and time-point [T1 and T2] and a between-subjects factor of schooling group [FG and KG] was run on the beta coefficient brain maps using the *3dLME* function in AFNI. Brain maps showing the interactions were thresholded at a voxel-wise threshold on *p* < .01 at 175 voxels using *3dClustSim* and *3dClusterize*. To meet the objectives of the current study, we focussed on two interactions: (1) chromophore x schooling-group x time-point, and (2) chromophore x schooling-group x time-point x load. Note that while trial type and accuracy were modelled, we did not include these variables in the interactions of interest due to a lack of trials at each load.

### 2.10 Outlier correction

All behavioural data (accuracy, capacity, vocabulary, and numeracy) were screened for outliers. The Mahalanobis distance (MD) method was used to identify longitudinal outliers (alpha =0.001). In those participants who were identified as longitudinal outliers, data-points at each of the time-points were also checked to see if they fell outside of ± 3 SDs from the mean. Data points from three participants were identified as outliers (2 participants for accuracy and one participant for vocabulary). Thus, data from these three participants were excluded from all modelling analyses.

### 2.11 Modelling framework

Behaviour and brain activation data were fitted with latent change score (LCS) models (Kievit et al., 2018) to investigate if there was an effect of transition from kindergarten to primary school. First, a series of univariate LCS models were fitted to each of the variables to investigate the degree of change within each domain. Next, univariate LCS models of variables that showed an effect of transition from kindergarten to primary school were extended into bivariate LCS models by adding an extra domain. This bivariate extension allows us to investigate the extent to which change in one domain (i.e., WM) is a function of the starting point in another domain (i.e., vocabulary), or vice versa, or bidirectional. Models were estimated in the lavaan software package in R version 3.6.2 (Rosseel, 2012). More details of the modelling framework are provided in the Supplementary materials.

## 3 Results

### 3.1 Univariate LCS modelling

We constructed separate univariate models for accuracy, capacity, HbO concentration, HbR concentration, vocabulary scores, non-symbolic and symbolic numeracy score. Age and gender were included as covariates. First, we investigated whether there was a significant change across time-points in FG and KG children. Next, we tested whether this change across time-points differed between FG and KG children to tease apart the schooling-related effects from age-related effects. For capacity, accuracy, vocabulary scores and numeracy scores, we categorized an effect as being associated with the impact of schooling (above and beyond age-related effects) if improvement in FG children from T1 to T2 significantly differed from change (either improvement or deterioration from T1 to T2) in KG children. For brain data, we categorized an effect as being associated with the impact of schooling (above and beyond age-related effects) if change in FG children significantly differed from change in KG children.

#### 3.1.1 Accuracy

Mean and SEM for the parameter estimates are shown in Supplementary Table 1. FG children showed a significant increase in accuracy from T1 and T2, while KG children did not – see Fig. 2b. Constraining the change to be equal across groups led to a significant drop in model fit, $\Delta x^{2}=9.66,\;\Delta df=1,\;p=.002$, confirming that the change in accuracy from T1 to T2 differed across groups. Further, constraining baseline scores at T1 to be equal across groups also did not lead to a significant drop in model fit $\left(\Delta x^{2}=2.42,\;\Delta df=1,\;p=.12\right)$ suggesting that both groups had comparable accuracy values at T1. Thus, *only* FG children showed an increase in accuracy from T1 to T2 suggesting that exposure to the first year of formal schooling conferred benefits to accuracy.

#### 3.1.2 Capacity

Mean and SEM for the parameter estimates are shown in Supplementary Table 1. Both FG and KG showed a significant increase in capacity from T1 to T2 – see Fig. 2a. However, there was no significant drop in model fit when change in capacity was constrained to be equal across groups $\Delta x^{2}=0.2,\;\Delta df=1,\;p=.88$. Additionally, there was no significant drop in model fit when values at T1 were constrained to be equal across groups $\Delta x^{2}=2.50,\;\Delta df=1,\;p=.11$. Thus, both groups of children only showed age-related improvements in capacity. Exposure to the first year of schooling did not improve WM capacity.

#### 3.1.3 Brain activation

**Fig. 3:**
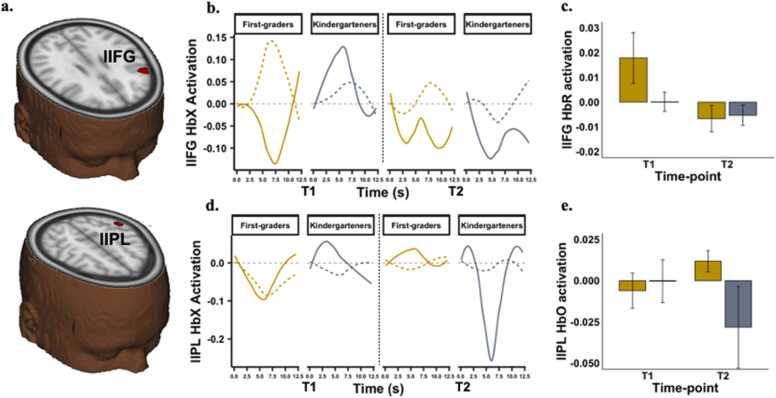
(a) Significant interaction between chromophore, schooling group and time-point at left inferior frontal gyrus (lIFG – Centre of mass MNI coordinates: 49.7, −21.2 and 27.5, volume: 188 voxels) and left inferior parietal lobule (lIPL – Centre of mass MNI coordinates: 53.2, 44.2 and 48.9, volume: 185 voxels). Average HbO and HbR concentration time-series plot for (b) lIPL and (c) lIFG. HbO concentration is shown in bold lines and HbR concentration is shown in dashed lines. FG are shown in yellow and KG are shown in grey. (d) Mean ± SEM HbO beta estimate for lIPL. (e) Mean ± SEM HbR beta estimate for lIFG.

From the linear mixed effect modelling applied to the brain data, only the interaction between chromophore, schooling-group and time-point was significant. The interaction between chromophore, schooling-group, time, and load was not significant. The significant interaction between chromophore, schooling-group and time-point was observed in two brain regions – left inferior frontal gyrus (lIFG – Centre of mass MNI coordinates: 49.7, −21.2 and 27.5, volume: 188 voxels) and left inferior parietal lobule (lIPL – Centre of mass MNI coordinates: 53.2, 44.2 and 48.9, volume: 185 voxels) – see Fig. 3a. For each significant cluster, activation was averaged across voxels, load, and trial type so that there was an averaged value per chromophore (HbO/HbR) and time (T1 and T2) for each participant. These averaged HbO and HbR values were inserted into the modelling analyses discussed in later sections. Note that since HbR and HbO concentrations are inversely related, we interpret a significant increase in HbO concentration or a significant decrease in HbR concentration as *engagement* of a cortical area, and significant decrease in HbO concentration or a significant increase in HbR concentration as *reduced engagement* or *suppression*.

##### 3.1.3.1 HbO concentration

Mean and SEM for the parameter estimates for the two clusters are shown in Supplementary Table 2.

Fitting a separate model to lIFG HbO concentration revealed that neither FG nor KG showed a significant change from T1 to T2. Constraining the change from T1 to T2 to be equal across groups also did not lead to a significant drop in model fit $\Delta x^{2}=3.57$, Δdf=1, p=.06. Thus, there were no schooling-related effects on lIFG HbO concentration.

In the univariate model fitted to lIPL HbO concentration, neither FG nor KG children showed a significant change in concentration from T1 to T2. However, constraining the change from T1 to T2 to be equal across groups led to a significant drop in model fit, $\Delta x^{2}=6.33$, Δdf=1, p=.01. Further, constraining T1 estimates to be equal across groups did not lead to a significant drop in model fit (${∆\text{x}}^{2}\text{ = }2.40\text{, }∆\mathrm{df}\;=\;1,\;p\text{ =.}12\text{)}$. Our results showed that the two groups differed in *how* they changed from T1 to T2, i.e., the group difference in change in lIPL HbO concentration was driven by FG showing a trend for greater concentration at T2 compared to T1, and conversely, KG showing a trend for lesser concentration at T2 compared to T1 (Fig. 3c and d). To summarize, exposure to the first year of schooling was associated with greater lIPL engagement.

##### 3.1.3.2 HbR concentration

Mean and SEM for the parameter estimates for the two clusters are shown in Supplementary Table 3.

Fitting a univariate model to lIFG HbR concentration revealed that FG children showed a significant decrease in concentration from T1 to T2 while KG children did not – see Fig. 3d and e. Constraining the change to be equal across groups lead to a significant drop in model fit, ${∆\text{x}}^{2}\text{ = }4.63\text{, }∆\mathrm{df}\;=\;1,\;p\text{ =.}031$. Further, constraining concentration at T1 to be equal across the groups did not lead to a significant fit in model fit, ${∆\text{x}}^{2}\text{ = }1.12\text{, }∆\mathrm{df}\;=\;1,\;p\text{ =.}29$. Thus, exposure to the first year of schooling resulted in greater lIFG engagement, over and above age-related effects.

In the univariate model fitted to lIPL HbR concentration, neither FG nor KG showed a significant change in concentration from T1 to T2. Further, constraining change to be equal across groups did not lead to a significant drop in model fit, $\Delta x^{2}=.24,\;\Delta df=1,\;p=.62$. Thus, there were no schooling-related effects on lIPL HbR concentration.

Taken together, our brain findings reveal that exposure to the first year of formal schooling was associated with greater engagement of left-lateralized IPL and IFG.

#### 3.1.4 Vocabulary

**Fig. 4:**
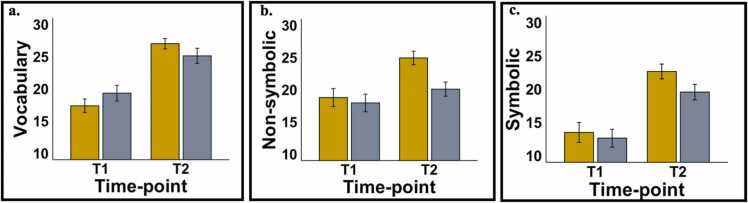
(a) Vocabulary scores, (b) Non-symbolic numeracy scores and (c) Symbolic numeracy scores across time-points. FG are shown in yellow and KG are shown in grey.

Mean and SEM for the parameter estimates for vocabulary scores are shown in Supplementary Table 4. Both FG and KG showed a significant increase in scores from T1 to T2. Constraining the change in both groups to be equal also led to a significant drop in model fit $∆{\text{x}}^{2}\text{=}4.48\text{, }∆\mathrm{df}\;=\;1,\;p\text{ =.}034$, suggesting that FG demonstrated a greater improvement in scores compared to KG (see Fig. 4a). Further, no significant changes in model fit were found after constraining T1 estimates to be equal across groups $\left(\Delta x^{2}=.01,\;\Delta df=1,\;p=.91\right)$. Thus, both groups of children showed comparable improvements in vocabulary scores from T1 to T2. However, FG children showed greater improvements compared to KG children suggesting that exposure to the first year of formal schooling conferred additional benefits to building vocabulary knowledge, over and above age-related effects.

#### 3.1.5 Numeracy

Mean and SEM for the parameter estimates for non-symbolic and symbolic scores are shown in Supplementary Table 4.

For non-symbolic numeracy, FG showed a significant change from T1 to T2 while KG did not – see Fig. 4b. When the change was constrained to be equal across groups, there was a borderline non-significant drop in model fit ($∆{\text{x}}^{2}\text{ = }3.49\text{, }∆\mathrm{df}\;=\;1,\;p\text{ =.}06\text{)}$. Further, no significant changes in model fit were found after constraining T1 estimates to be equal across groups ($∆{\text{x}}^{2}\text{ =.}13\text{, }∆\mathrm{df}\;=\;1,\;p\text{ =.}72\text{)}$. In summary, only FG children showed a significant improvement in non-symbolic numeracy scores from T1 to T2. Thus, exposure to formal schooling was associated with a borderline improvement in non-symbolic numeracy scores, over and above age-related effects.

For symbolic numeracy, both FG and KG showed a significant increase in scores from T1 to T2 – see Fig. 4c. However, no significant changes in model fit were found when the change was constrained to be equal across groups ($∆{\text{x}}^{2}\text{ = }1.02\text{, }∆\mathrm{df}\;=\;1,\;p\text{ =.}31\text{)}$. Thus, exposure to schooling did not confer any additional advantage to improving symbolic numeracy scores, over and above age-related effects.

In summary, our results revealed that exposure to the first year of formal schooling was associated with improvement in accuracy, greater engagement in left-lateralized IPL and IFG and an improvement in vocabulary and non-symbolic numeracy scores, over and above effects of increasing age.

### 3.2 Bivariate LCS modelling

We constructed bivariate models to investigate whether there was any association between schooling-related improvements observed in accuracy, brain function, vocabulary, and non-symbolic numeracy scores in FG children. Six bivariate models were constructed between: (1) accuracy and vocabulary scores, (2) lIPL HbO concentration and vocabulary scores, (3) lIFG HbR concentration and vocabulary scores, (4) accuracy and non-symbolic numeracy scores, (5) lIPL HbO concentration and non-symbolic numeracy scores, and (6) lIFG HbR and non-symbolic numeracy scores. Estimates for bivariate couplings for significant models are shown in Supplementary Tables 5 and 6.

#### 3.2.1 Accuracy and vocabulary scores

Accuracy at T1 predicted improvement in vocabulary from T1 to T2 (β = 34.58, *p* = .01). Further, constraining the coupling between average A′ at T1 and improvement in vocabulary to zero resulted in a significant drop in model fit ($∆{\text{x}}^{2}\text{ = }9.83\text{, }∆\mathrm{df}\;=\;1,\;p\text{ =.}002\text{)}$. No other coupling estimates were significant. Thus, children who had better WM performance before they started formal schooling showed greater improvement in vocabulary scores after one year of schooling.

#### 3.2.2 lIPL HbO concentration and vocabulary scores

No coupling estimates were significant in this model. Thus, there was no association between lIPL engagement and vocabulary scores.

#### 3.2.3 lIFG HbR concentration and vocabulary scores

Activation at T1 did not predict improvement in vocabulary scores from T1 to T2 (β = −0.01, *p* = .87). However, there was a significant covariance between change in lIFG HbR activation and improvement in vocabulary scores (β = −4.26, *p* = .047). Further, constraining the coupling between change in HbR concentration and change in vocabulary to zero resulted in a significant drop in model fit ($∆{\text{x}}^{2}\text{ = }9.51\text{, }∆\mathrm{df}\;=\;1,\;p\text{ =.}002\text{)}$. No other coupling estimates were significant. Thus, following one year in school, children who showed greater change in lIFG engagement demonstrated greater improvements in vocabulary scores.

#### 3.2.4 Accuracy and non-symbolic numeracy scores

No coupling estimates were significant. Thus, there was no association between accuracy and non-symbolic numeracy scores.

#### 3.2.5 lIPL HbO concentration and non-symbolic numeracy scores

lIPL HbO concentration at T1 predicted improvement in non-symbolic numeracy scores from T1 to T2 (β = 0.42, *p* < .001). Further, constraining the coupling between lIPL HbO concentration at T1 and improvement in non-symbolic numeracy scores to be zero led to a significant drop in model fit ($∆{\text{x}}^{2}\text{ = }15.46\text{, }∆\mathrm{df}\;=\;1,\;p\text{ =.}001\text{).}$No other coupling estimates were significant. Thus, children who showed greater lIPL engagement at the start of formal schooling demonstrated greater improvement in non-symbolic numeracy scores after one year in school.

#### 3.2.6 lIFG HbR activation and non-symbolic numeracy scores

No coupling estimates were significant. Thus, there was no association between lIFG HbR concentration and non-symbolic numeracy scores.

To summarize, after a year in school, improvement in vocabulary scores was predicted by WM performance prior to starting school and increased left-lateralized IFG engagement across the year in school. Further, improvement in non-symbolic numeracy scores after one year in school was predicted by engagement of left-lateralized IPL prior to starting school.

## 4 Discussion

The current study employed a modified school cut-off design to investigate the impact of the first year of primary school on WM function and academic abilities beyond age-related developmental improvements. Our first finding revealed that one year in formal schooling was accompanied by an improvement in WM performance and greater engagement of left-lateralized fronto-parietal network. Second, exposure to a year in formal schooling also led to greater improvement in vocabulary scores and non-symbolic numeracy scores. Finally, we found that improvements in vocabulary and non-symbolic numeracy scores following one year in primary school was predicted by WM function.

### 4.1 One year in formal schooling is associated with improvement in WM function

The transition from kindergarten to formal schooling was associated with an improvement in WM performance and greater engagement of left-lateralized IPL and IFG. Our findings are consistent with evidence showing that developmental improvements in verbal and visuospatial WM measured in children in Grade 1 might be linked to time spent in the classroom (Roberts, 2015). Further evidence suggests that children showed greater improvement in WM skills during school-year months compared to summer months suggesting that a structured learning environment was important for WM development (Finch, 2019). A structured instructional environment places heavy emphasis on reading, writing and effective communication skills. For instance, school children are expected to learn how to follow a timetable and strict routines and manage their time and resources effectively (Sink et al., 2007). Further, school classrooms hold more children, compared to kindergarten, thus, children need to exert greater executive control to adapt to group-based learning approaches without succumbing to distraction. Exposure to this disciplined learning environment in school might enhance cortical specialization in regions like lIPL and lIFG, involved in integrating verbal, semantic, phonological processes with WM function. Concretely, left-lateralized IPL is involved in verbal WM processing (Nagel et al., 2013), orienting visuo-spatial attention (Corbetta et al., 2000, Culham and Kanwisher, 2001), top-down biasing to foreground items using semantic and conceptual details (Nee et al., 2011, Nee and Jonides, 2008) and integration of featural and semantic information to form a coherent concept (Chou et al., 2009). Along a similar vein, left-lateralized IFG is involved in executive control to select between competing representations, maintenance, refreshing and resisting proactive interference (Nee, 2013, Nelson et al., 2009, Zhang et al., 2004), and WM processing during semantic and phonological tasks (Liakakis and Nickel, 2011). Drawing upon this evidence, we suggest that FG children might routinely boost left-lateralized fronto-parietal engagement to integrate verbal instructions with maintaining complex information. They might also adopt efficient visuospatial strategies to regulate and manage greater demands on executive control to meet set goals and tasks.

### 4.2 One year in formal schooling is associated with improvement in academic abilities

Exposure to one year of schooling was linked to improvement in vocabulary scores, beyond developmental changes. This finding agrees with more general evidence linking improvements in literacy to the transition from kindergarten to primary school (Varnhagen et al., 1994, Christian et al., 2000, Morrison et al., 1995, Kim and Morrison, 2018, Ferreira and Morrison, 1994). Children are exposed to different types of interactions in school, compared to kindergarten. In school, children interact with teachers and older peers in different grades during group activities, recess, lunchrooms, and in the playground. During these interactions, children might learn to accumulate richer word knowledge and form complex sentence structures. There are also differences in instruction between the learning environments in kindergarten and primary school. Concretely, in primary school, teachers emphasize more rote learning and adopt traditional approaches which emphasize memorization of content compared to kindergarten teachers who might engage in play-oriented and more constructivist approaches to learning (Uibu et al., 2011). Instruction might be linked to children’s vocabulary acquisition with high-intensity instruction (e.g., in-depth discussion) facilitating learning of around 42% of taught words and low-intensity instruction (e.g., giving definitions) facilitating a further 22%.

One year in primary school was also associated with an improvement in non-symbolic numeracy scores. More generally, this finding is consistent with previous work assessing number recognition, counting, cardinality, subtraction and addition in school children (Christian et al., 2000, Morrison et al., 1997). Within the context of the current study, it is possible that, in schools, children are expected to repeatedly perform magnitude comparisons and numerical operations quickly and efficiently – resulting in better scores. Alternatively, it is also possible that high-quality instruction within a structured learning environment might improve overall visuo-spatial attention and WM processing, resulting in transferable skills for performing magnitude comparisons. Interestingly, however, the benefits afforded to FG children on non-symbolic numeracy abilities did not transfer to symbolic numeracy scores – both groups performed comparably. A potential explanation for this finding is that *all* children might be equally exposed to symbolic representations through learning activities and games (Li et al., 2018). Thus, there might be no real advantage that exposure to primary schooling can confer upon symbolic numeracy abilities. However, more research in this domain is necessary to investigate if this finding is replicable with non-symbolic and symbolic numerals larger in value than those used in the numeracy screener of the current study.

### 4.3 Improvement in academic abilities following a year in primary school is linked to WM function

We reported that better WM performance prior to starting school predicted greater improvement in vocabulary scores following the year in primary school. This finding is in line with previous evidence associating WM performance and vocabulary in children (Engel de Abreu and Gathercole, 2012, Verhagen and Leseman, 2016, Kim, 2015, Vlach and DeBrock, 2017). Adams and Gathercole (2000) reported that 4-year-old children who had better memory abilities produced longer utterances of spoken language that contained more unique words. Nevo and Bar-Kochva (2015) investigated the predictive effects of early WM abilities on later developing reading skills, by testing WM in kindergarten and reading skills in grades 1, 2, and 5. They found that memory measures were correlated with reading until grade 5. Further, visuo-spatial memory predicted reading comprehension in grades 2 and 5, alluding to a long-lasting role for early WM as a predictor of variance in reading.

How might WM function *prior* to attending school link to vocabulary development? Evidence from infancy and toddlerhood studies demonstrate support for the role of WM function in word acquisition. Early in development, retention of name-object associations is enhanced by visual familiarity with objects (Clerkin et al., 2017, Kucker and Samuelson, 2012, Fennell, 2012). Young children constantly try to reach out, hold and manipulate objects resulting in dominant WM representations of those objects in their field of view. If labels of these objects are used alongside maintenance of object representations in WM, children learn these associations (Yu and Smith, 2012). Thus, the quantity, persistence, and quality of rich visuo-haptic-verbal experiences throughout early development can be critical for acquisition and development of word knowledge.

We also found that greater lIFG engagement during the year in school was associated with greater improvement in vocabulary scores. Exposure to more complex instruction and structured learning in school might require children to simultaneously refine and specialize brain networks subserving both WM processes and comprehension, language, and speech processes. Within this context, left-lateralized IFG has notable significance because it is involved in both effortful control and language comprehension and production. We suggest that in school, children might be actively applying executive control and WM processing to refine word acquisition and production.

Our findings revealed that FG children who showed greater lIPL engagement prior to starting school demonstrated greater improvement in non-symbolic numeracy scores following a year in school. Previous studies have shown that IPL is actively involved in number and calculation tasks (Arsalidou et al., 2018, Bugden et al., 2012, Dehaene et al., 2003, Fehr et al., 2007, Haist et al., 2015, Kesler et al., 2011, Menon et al., 2000, Zhang, 2019). In children, greater lIPL engagement is observed during calculation tasks, implying the need to coordinate more complex, relational and figurative schemes using mental attention capacity (Arsalidou et al., 2018). Dehaene and colleagues parcellated the parietal cortex into three distinct functional circuits based on previous studies on mathematical neurocognition – a horizontal section of the intraparietal sulcus responsible for nonverbal representation of numerical quantity, left angular gyrus overlapping with the language system, responsible for verbal coding of numbers, and bilateral posterior superior parietal lobule involved in visuo-spatial attention orienting and working memory (Dehaene et al., 2003). In the current study, FG children might have activated all three systems. For instance, children might have relied on verbal coding, refreshing, and updating of WM representations while counting the dots on each side of the display. They might have also relied on visuo-spatial attention to grossly estimate magnitude differences.

The evidence presented in previous sections implies that vocabulary and numeracy development might rely on both visuo-spatial and verbal processing. Yet, in the current study, these academic abilities were associated with behavior/brain function on the *color* change detection task that only uses simple colored stimuli. An explanation for this association is that children might have relied on both visual and verbal processing pathways to successfully hold items in WM. For example, children might have held the visual properties of colors in visual WM and/or held the labels for colors in verbal WM. Given the uncertainty in conjunction and disjunction between visual and verbal WM pathways in development, future work should also specifically investigate how transition from kindergarten to formal schooling impacts different types of WM and critically, whether specific WM processes might be more related to some academic abilities.

The association between WM processing prior to starting school and later academic abilities is supported by evidence from studies on school-readiness. Swayze and Dexter (2018) found that WM in pre-schoolers was predictive of school readiness assessed through abilities of language, emergent literacy skills, mathematical literacy and cognitive development/intelligence (Swayze and Dexter, 2018). In another study, classroom engagement, number knowledge and receptive vocabulary were predicted by WM scores measured as early as toddlerhood (Fitzpatrick and Pagani, 2012). Taken together, collective findings imply that specific abilities present prior to starting school might better equip children for academic success.

## 5 Limitations

We created a representative template head model for each cap size by digitizing scalp landmark and source and detector positions from one child for each cap size. In future studies, variance due to changes in head shape, head size and probe placement across participants and across time-points should be accounted for by using digitisations from each individual child.

## 6 Implications and future work

In the current study, we found that formal schooling improved WM function, vocabulary knowledge and non-symbolic numeracy. Further, while vocabulary and numeracy scores did not worsen in KG, who stayed in kindergarten for another year, there was a trend for decreased WM accuracy. Thus, at least at the end of the first year in school, our findings suggest that an early enrolment does not *stagnate* or *worsen* WM function and academic abilities. These findings might be helpful for informing pedagogical practices/measures that can be put in place to smoothen the transition between kindergarten and formal schooling for all children, regardless of whether they are enrolled as soon as they become eligible or after they are deferred in kindergarten for another year. However, more investigation is necessary to ascertain whether neurocognitive function and academic abilities in KG children ‘catch up’ to FG children following a year after their own enrolment in school. Our project envisioned a third time-point of data collection from both groups; however, we could not pursue this goal due to the impact of COVID-19 restrictions on school attendance, home visits, study design, methodology and hypotheses.

## 7 Conclusions

The current study employed a modified school cut-off design to tease apart the impact of exposure to one year of schooling from age-related maturational changes on WM processing and academic abilities. We followed and compared two groups of children who were similar in age but differed in their experience of the schooling environment. We found that exposure to one year of formal schooling resulted in an improvement in WM performance and greater engagement in lIPL, an area important for managing and integrating visuo-spatial attention with semantic and conceptual information, and lIFG, an area involved in executive control and language processing. Exposure to primary school was also associated with an improvement in vocabulary scores and non-symbolic numeracy scores. Critically, in children who had the exposure to formal schooling, WM function predicted improvement in vocabulary and non-symbolic numeracy scores. Our findings contribute to a growing body of literature closely examining the neurocognitive and academic benefits conferred to children after exposure to a structured formal schooling environment.

## Funding sources

The project was funded by a Jacobs Foundation Research Fellowship to Y. L. Shing (JRF 2018–2020) and by a match-funded Ph.D. studentship from the University of Stirling. The work of S. Wijeakumar was supported by funding from the Bill and Melinda Gates Foundation (OPP1119415) and the 10.13039/501100000275Leverhulme Trust (RPG-2019-286). The work of Y. L. Shing was also supported by the 10.13039/501100000780European Union (ERC-2018-StG-PIVOTAL-758898), the Deutsche Forschungsgemeinschaft (10.13039/501100001659German Research Foundation, Project-ID 327654276, SFB 1315, “Mechanisms and Disturbances in Memory Consolidation: From Synapses to Systems”), and the Hessisches Ministerium für Wissenschaft und Kunst (HMWK; project 'The Adaptive Mind').

## Author contributions

Conceptualization: YLS, SW, ER and CAM; Methodology: YLS, CAM and SW; Formal analysis: CAM, CD, SW and YLS; Data collection: CAM and CD; Writing: CD, CAM, ER, YLS and SW.

## Declaration of Competing Interest

The authors declare that they have no known competing financial interests or personal relationships that could have appeared to influence the work reported in this paper.

## Data Availability

All group-level data and analysis scripts will be made publicly available via the Open Science Framework before publication is finalized. As an example from another article from the same project, see https://osf.io/nyjqe/.
