# Supplementary material for: The first year in formal schooling improves working memory and academic abilities
Source: Dev Cogn Neurosci. 2023 Jan 29;60:101205. doi: 10.1016/j.dcn.2023.101205 (PMC9898018; doi:10.1016/j.dcn.2023.101205)
Supplement: Supplementary file 1 — Supplementary material [file mmc1.docx]

**Supplementary Materials.**

10.1 Socioeconomic status and socio-emotional behaviours.

Parents filled in a socioeconomic scale that assessed educational qualifications and annual income, and Strengths and Difficulties questionnaire ^28^ that assessed children’s socio-emotional behaviours. To inquire whether there were any measurable differences in family socioeconomic status or socio-emotional behaviours between FG and KG children at T1, categorical data from both questionnaires were log-transformed and an independent samples t-test was carried out on each variable of the questionnaires. Parents reported attaining an undergraduate, postgraduate or a doctorate degree and had reported annual income between £30,000 - £50,000. There was no significant difference between groups in parental educational attainment and parental annual income (*p* > .05). Further, there was no significant difference between groups in parent-reported measures of emotional problems, conduct problems, hyperactivity, peer problems, and prosocial behaviours (*p* > .05).

10.2 Modelling Framework*.*

Behaviour and brain activation data were fitted with latent change score (LCS) models to investigate if there was an effect of transition from kindergarten to primary school. First, a series of univariate LCS models were fitted to each of the variables to investigate the degree of change within each domain. The model was set-up as a multi-group model (the same model was fitted separately to the two groups) allowing for key parameters to be estimated independently for each group. The basic model equation is as follows:

$$X_{i,t2}= X_{i,t1}+ {\Delta X}_{i,1}$$

where an individual’s score *(i)* on a domain *(X)* at time-point 2 *(t2)* is equal to the sum of the individual’s score in that domain at time-point 1 *(t1)* and the change, or difference score (${\text{∆}\text{X}}_{\text{i}}$). By fixing the autoregressive parameter between T1 and T2 to 1, we assume that intervals are equidistant across individuals ^71^. The change score equation can be modified to:

$${\Delta X}_{i,1}= X_{i,t2}- X_{i,t1}$$

Thus, the LCS factor (${\text{∆}\text{X}}_{\text{i}}$) is measured by T2, with a factor loading fixed to 1. The change between T1 and T2 is captured by the mean of the LCS factor, while the extent to which individuals differ in the amount of change is captured by the variance. Finally, a covariance or regression parameter *(*$\text{β)}$ is added to the change score, to determine whether the amount of change depends on scores at T1:

$${\Delta X}_{i,1}= \beta*X_{i,t1}$$

Thus, this model provides three important pieces of information: (i) whether there is significant average change per schooling group across time-points, as determined by (${\text{∆}\text{X}}_{\text{i}}$), (ii) whether individuals differ in how much they change, as determined by the variance, and (iii) whether the change is dependent on scores at T1. Importantly, as there are as many unique pieces of information entering the model as parameters to be estimated, the basic model is just-identified. The key questions can be investigated by employing parameter constraints. For the current study, the interest lies in whether the two groups (FG and KG) differed in the amount of change they demonstrated in a specific domain (i.e., WM) across time-points. For this, a free model, where the change in both groups can differ, and a constrained model, where the change is constrained to be equal, can be specified. If the constrained model leads to a significant drop in model fit (as indexed by the difference in chi-square test), this will indicate that the two groups differ in their amount of change.

Finally, this univariate LCS model was extended into a bivariate LCS model by adding an extra domain. The new equation is as follows:

$${\Delta X}_{i,1}= \beta1*X_{i,t1}+c*Y_{i,t1}$$

where the change scores of two domains (X and Y) are a function of two processes: the self-feedback parameter ("β)” and a coupling process (c). With such bivariate model, we gain four additional pieces of information, (1) whether the scores in X are associated with the scores in Y at T1 (intercept covariance), (2) whether the change in X is associated with the change in Y (change covariance), (3) whether the change in X is a function of the starting point of Y (coupling_y->x_), and (4) whether the change in Y is a function of the starting point of X (coupling_x->y_). Thus, this bivariate extension allows us to investigate the extent to which change in one domain (i.e., WM) is a function of the starting point in another domain (i.e., vocabulary), or vice versa, or bidirectional.

Models were estimated in the lavaan software package in R (version 3.6.2, 2019; Rosseel, 2012). For both univariate and bivariate models, all HbO and HbR concentration was multiplied by 100 to adjust for scaling when reporting statistics. Full information maximum likelihood was used to handle missing data, allowing data from all participants to be included for analysis. Relative change in model fit was assessed using the chi-square difference test.


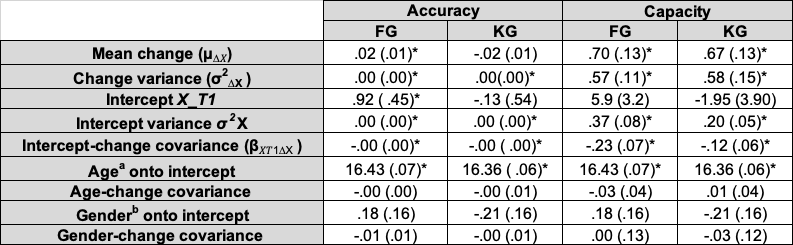


Supplementary Table 1. Parameter estimates for univariate models of accuracy and capacity.

Standard error is in parantheses.

*Asterisks denote significance at p< .05 level.

^a^Age = age in days / 100.

^b^Gender coded as 1 = girls, -1 = boys.


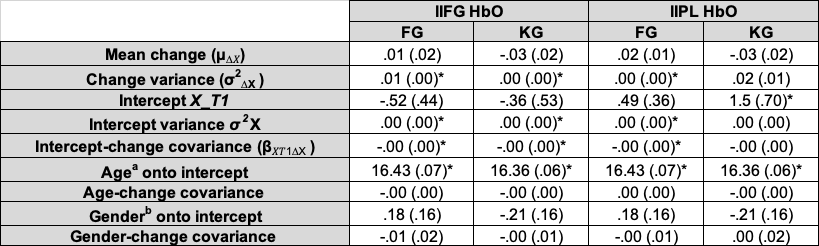


Supplementary Table 2. Parameter estimates for univariate models of HbO concentration in lIFG and lIPL clusters.

Standard error is in parantheses.

*Asterisks denote significance at p< .05 level.

^a^Age = age in days / 100.

^b^Gender coded as 1 = girls, -1 = boys.


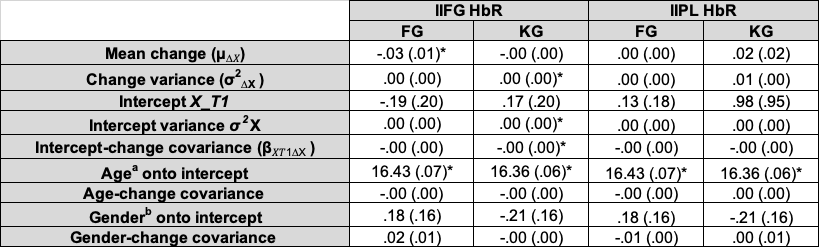


Supplementary Table 3. Parameter estimates for univariate models of HbR concentration in lIFG and lIPL clusters.

Standard error is in parantheses.

*Asterisks denote significance at p< .05 level.

^a^Age = age in days / 100.

^b^Gender coded as 1 = girls, -1 = boys.


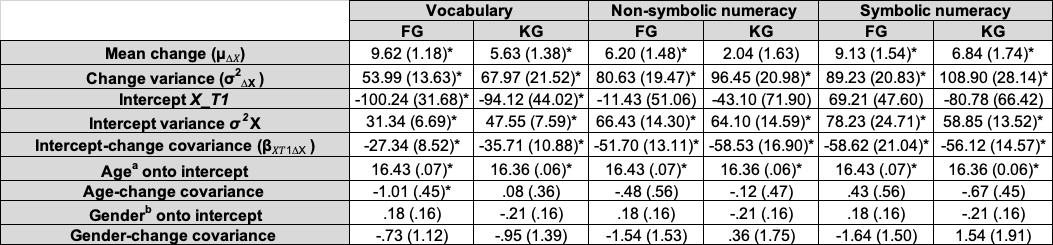


Supplementary Table 4. Parameter estimates for univariate models of vocabulary, non-symbolic numeracy, and symbolic numeracy scores.

*Asterisks denote significance at p< .05 level.

^a^Age = age in days / 100.

^b^Gender coded as 1 = girls, -1 = boys.


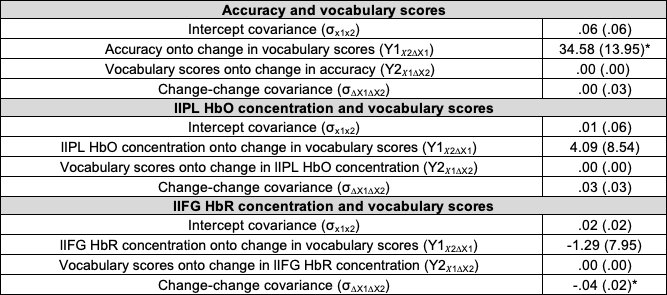


Supplementary Table 5. Bivariate models between accuracy and vocabulary scores, lIPL HbO concentration and vocabulary scores, and lIFG HbR concentration and vocabulary scores in FG children.

Standard error are in parantheses.

*Asterisks denote significance at p< .05 level.


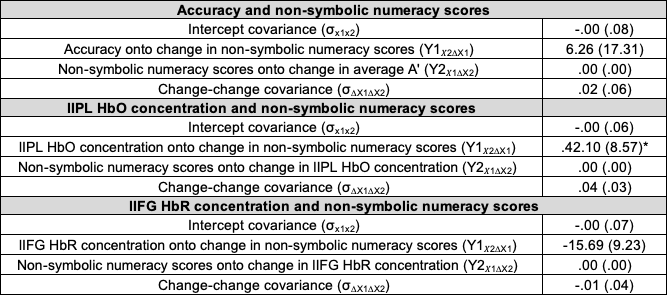


Supplementary Table 6. Bivariate models between accuracy and non-symbolic numeracy scores, lIPL HbO concentration and non-symbolic numeracy scores, and lIFG HbR concentration and non-symbolic numeracy scores in FG children.

Standard error are in parantheses.

*Asterisks denote significance at p< .05 level.
